# A Study of Dielectrophoresis-Based Liquid Metal Droplet Control Microfluidic Device

**DOI:** 10.3390/mi12030340

**Published:** 2021-03-23

**Authors:** Lu Tian, Zi Ye, Lin Gui

**Affiliations:** 1 Beijing Smart-Chip Microelectronics Technology Company, Ltd., Beijing 100192, China; tianlu1@sgitg.sgcc.com.cn; 2 Key Laboratory of Cryogenics, Technical Institute of Physics and Chemistry, Chinese Academy of Sciences, 29 Zhongguancun East Road, Haidian District, Beijing 100190, China; yezi_1992@mail.ipc.ac.cn; 3 School of Future Technology, University of Chinese Academy of Sciences, Beijing 100039, China

**Keywords:** dielectrophoresis, microfluidics, metal droplet driving

## Abstract

This study presents a dielectrophoresis-based liquid metal (LM) droplet control microfluidic device. Six square liquid metal electrodes are fabricated beneath an LM droplet manipulation pool. By applying different voltages on the different electrodes, a non-uniform electric field is formed around the LM droplet, and charges are induced on the surface of the droplet accordingly, so that the droplet could be driven inside the electric field. With a voltage of ±1000 V applied on the electrodes, the LM droplets are driven with a velocity of 0.5 mm/s for the 2.0 mm diameter ones and 1.0 mm/s for the 1.0 mm diameter ones. The whole chip is made of PDMS, and microchannels are fabricated by laser ablation. In this device, the electrodes are not in direct contact with the working droplets; a thin PDMS film stays between the electrodes and the driven droplets, preventing Joule heat or bubble formation during the experiments. To enhance the flexibility of the chip design, a gallium-based alloy with melting point of 10.6 °C is used as electrode material in this device. This dielectrophoresis (DEP) device was able to successfully drive liquid metal droplets and is expected to be a flexible approach for liquid metal droplet control.

## 1. Introduction

Droplet-based microfluidics, also known as “digital microfluidics”, has become a hot spot in the chemical and biological sciences [1,2], benefitting from its advantages, such as no cross-contamination, small sample size, and potential of high throughput analysis [3]. Droplet manipulation is the base of digital microfluidics in which the droplet control is of most importance. Approaches of droplet control can usually be categorized as active and passive methods [4], where passive control is of less interest due to its lack of flexibility. Among all active manipulation methods, electric-based ones are most popular because there are diverse and convenient ways to apply the electrical field inside microchannels. Normal electrical active droplet driving methods include electrostatic-charging actuation [5,6], electrowetting-on-dielectric (EWOD) [7], and dielectrophoresis (DEP) [8,9].

In most droplet-based microfluidics studies, droplets are often made of deionized water or aqueous solution and flow inside the oily or gaseous medium, with cells [10], particles [11], or multiphase droplets [12] wrapped inside. Recently, room temperature liquid metal (LM) has been introduced into droplet microfluidics as well [13]. Some metals, such as mercury, or alloys, most known as gallium-based alloys, have melting points lower than the room temperature and could form droplets at room temperature [14,15]. Among all these metal types, the gallium-based alloy is now of most interest due to its low toxicity, low cytotoxicity, and benign biocompatibility [16,17]. Droplets of this kind of metal have high electrical and thermal conductivities; hence, compared with ordinary aqueous solutions, they could perform different functions and applications. Due to their particular characteristics, the driving approaches are also slightly different. The most common principles of driving an LM droplet are electrowetting-on-dielectric and electrostatic. One of the most popular applications of LM droplet control is to use its high electrical and thermal conductivity in microfluidics as a micromechanical switch inside microchannels for thermal or electrical conduction [18,19,20]. Basic microfluidics components, such as micropumps [21] and mixers [22], are achieved by electrowetting (EW) controlled liquid metal droplets as well. Furthermore, Wu et al. [23] have recently developed a wheelchair mobile robot with electrical movement control of LM droplets, thereby largely expanding its application scope.

Among all the liquid metal droplet control studies mentioned above, DEP principle-based studies are barely reported. The DEP phenomenon is described as follows. If dielectric particles or droplets are put into non-uniform electric fields, these particles or droplets could move due to a force exerted from an electric field [24]. Instead of driving the droplet, DEP is commonly used in LM droplet generation [25]; however, DEP is also capable of driving LM droplets. In DEP microfluidics devices, the electric field and electrode material or design are of most concern, since the DEP’s driving effect largely depends on the electric field around the droplet. Standard electrode types include solid electrodes, deposition or sputtering electrodes, liquid electrodes, and salt bridge electrodes [26]. Room temperature liquid metal, as mentioned above, is a new choice of electrode material in microfluidics [27,28,29]. Compared with traditional solid electrodes, liquid electrodes have the advantages of easy fabrication, softness; and compared with salt bridge liquid electrodes, liquid metal has much a higher electrical conductivity. Unlike volatile and toxic mercury, gallium-based alloys have been widely used as electrodes in microfluidic devices because of their biocompatibility and safety.

Here, a new DEP-based LM droplet control microfluidic device is presented. An open channel structure is introduced into this device to simplify the manipulating process and waste recycling. A gallium-based alloy at room temperature (Ga_66_In_20.5_Sn_13.5_, melting point: 10.6 °C) is used as the electrode material. This device could drive liquid metal droplets moving between electrodes. Instead of the standard soft lithography process, laser ablation is used in this study to achieve simpler, faster, and cheaper microchannel fabrication. The LM droplet driving principle and the result with this DEP device is presented and discussed. 

## 2. Design and Methods

### 2.1. DEP Chip Design and Fabrication

**Figure 1:**
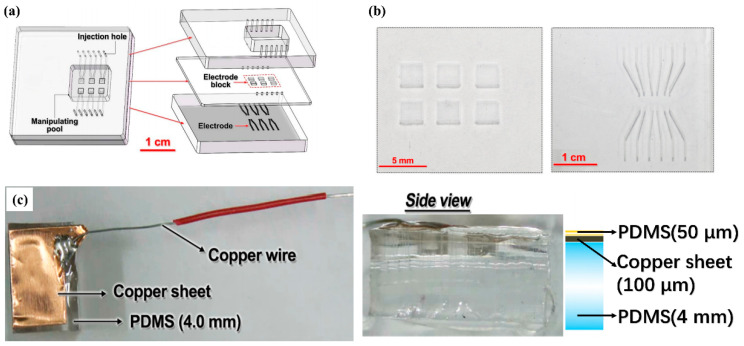
Design schematic and photo of the droplet driving device. (**a**) Three layers of the channel structure; (**b**) photo of the laser ablation channel; (**c**) photo of the copper sheet electrode.

Figure 1a shows the microchip structure of the DEP droplets control system. There are three layers in the microchip. The upper layer is 5.0 mm thick and has a rectangular through hole as the manipulating pool for the droplets. The middle layer is 600 μm thick and has six square island electrode paddles connected with the electrode microchannels of the lower layer. The lower layer is 5.0 mm thick and has six pairs of electrode microchannels. The upper and lower layers are determined to be 5.0 mm thick because they work as manipulation pool and bottom of the device, respectively. A thick PDMS block is stiff enough to support the whole device. The middle layer is thinner because the upper surface of this layer is in direct contact with the manipulated LM droplet, while voltages are applied on the square island electrode paddles, which are on the bottom side of the middle layer. The PDMS thickness between the electrode and the LM droplet could largely affect the electric field on the LM droplet, and finally determine the driving efficiency of this device. So, a thinner middle layer is chosen. These six pairs of microchannels are patterned and aligned with the six “island” electrode paddles in the middle layer. These six pairs of microchannels are used as the LM injection inlet and outlet of the island electrode paddles of the middle layer. The microstructures of the middle layer and lower layer are bonded face-to-face to make them connected with each other directly. Thus, the island electrode paddles in the middle layer and the paired microchannels in the lower layer are matched and connected to form six complete electrode channels, and work as six independent electrodes. In fabricating this two-layer structure alignment attention should be paid to making sure that each electrode paddle in the middle layer should be connected to the paired microchannel beneath it, and not to the one adjacent to it. As with the upper layer, the rectangular through cavity should cover all six complete electrode channels in the two-layer structure, i.e., both “island” electrode paddles and paired microchannels. Thus, the sidewalls of a rectangular cavity of the upper layer and the upper surface of the middle layer constitute the open area of the DEP device, working as the manipulation pool. When voltages are added to these electrodes, there is an inhomogeneous electric field generated in the manipulation pool, and the droplets there could be driven. For the convenience of the experimental operation, all the inlets and outlets of the microchannels are set and led out from the top, shown as through holes in Figure 1a. In the design, there is no direct contact between the working droplet and the electrodes, avoiding cross-contamination, electrode corrosion, and bubble formation during usage.

Fabrication of this DEP microchip included microchannel fabrication and the assembling process. Microchannels on both middle and lower layers are made by laser ablation. After drawing the designed channel structure with the L-edit software, the structure was imported to a laser ablation machine (Diatools, Shanghai, China), modeled LE-3, with laser power 1000 mW. An empty PDMS (10:1 mixture ratio by weight, Dow Corning, Midland, MI, USA) block, covered with black oily pigment to enhance the ablating strength, was then put under the laser beam. After calibrating and adjusting the beam position, the ablation began. After the ablation on the PDMS layer finished, residues should be wiped off along the microchannels to finish the microchannel fabrication process. The ablated channels are shown in Figure 1b. The “island” electrode paddles in the middle layer are squares with a side length of 2.0 mm, and the electrode channels in the lower layers are 200 μm in width. The height of all these channels is 80 μm. The upper layer was fabricated with a PDMS slide, where a rectangular area (1.3 cm × 1.5 cm) was fabricated by thrusting four thin copper sticks at its four corners first, and then cutting the sidelines with a knife. Finally, through holes were punched in the upper layer at all inlets and outlets of the electrode channels.

Assembling of this DEP microchip was achieved by the oxygen plasma bonding technology with a plasma cleaner (YZD08-2C, Yanzhao technology, Tangshan, China). The middle layer and lower layer were first aligned and bonded face-to-face as mentioned above, and then the two-layer structure was bonded with upper layer using the same bonding technique. Liquid metal electrodes were then made by directly injecting liquid metal (gallium-based alloy) into the electrode channels using a syringe. A silver-plated copper wire was put into the inlets and outlets of the electrode channels to connect the LM electrodes with the outer voltage supplier. All channel inlets and outlets were finally sealed with silicone rubber.

Because the liquid metal is easy to oxidize in the air, a layer of oxide film with high viscosity is formed on its surface and this film tends to adhere to PDMS. In order to avoid the liquid metal sticking to the bottom PDMS layer, a thin layer of insulating silicone oil was spread at the bottom of the manipulation pool for lubrication and insulation purposes.

### 2.2. Droplet Driving Experiment

The driving performance of LM droplets was tested with this DEP microchip. During the experiment, LM droplets were directly sucked up and dripped into the manipulation pool. The volume of the droplets used in the experiment was controlled by a pipettor. 

**Figure 2:**
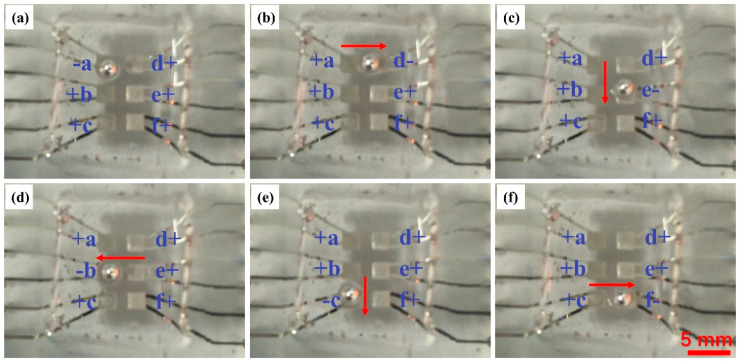
Experimental results for large liquid metal (LM) droplet driving. Six electrodes are numbered as a–d. “+” represents +1000 V, and “−” represents −1000 V. (**a**–**f**) Droplet position and travelling trace at different electrode conditions.

**Figure 3:**
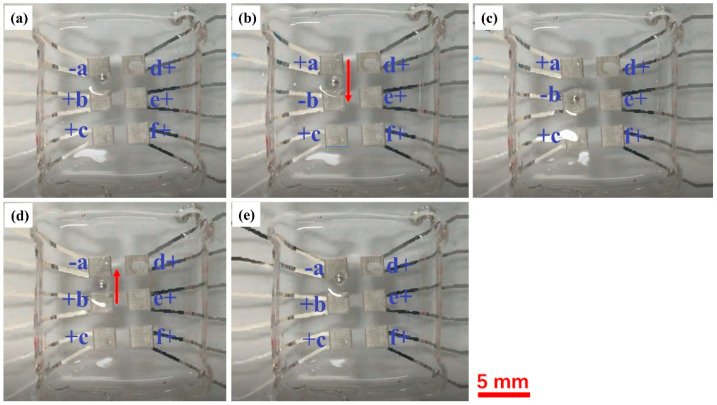
Experimental results for small LM droplet driving. Six electrodes are numbered as a–f. “+” represents +1000 V, and “−” represents −1000 V. The small droplet is controlled to travel between electrodes “a” and “b”. (**a**) Droplet initial position at electrode “a”; (**b**) droplet traveling towards electrode “b”; (**c**) stabilized position of droplet at electrode “b”; (**d**) droplet traveling back to electrode “a”; (**e**) stabilized position of droplet at electrode “a”.

**Figure 4:**
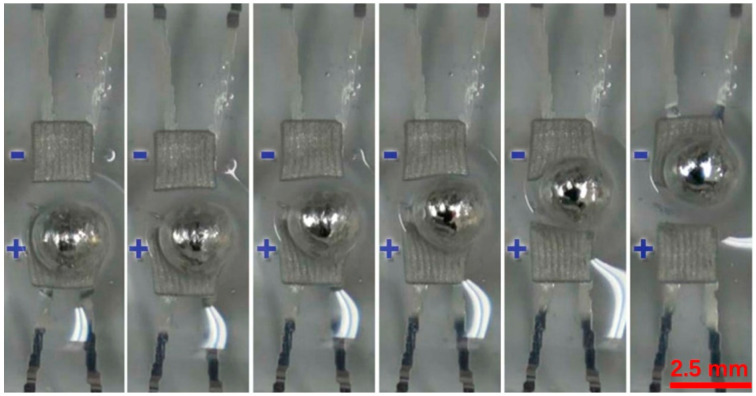
Details of large droplet moving from one electrode to another. Only two electrodes are included. “+” represents +1000 V, and “−” represents −1000 V.

The electric field during the experiment was realized by applying D.C. voltages on liquid metal electrodes in the microchip through a high voltage sequencer (HVS448 6000D, LabSmith, Inc., Livermore, CA, USA). Unless specified, the voltage applied during the experiment is ±1000 V and marked as “+” or “−” in Figure 2, Figure 3 and Figure 4. 

### 2.3. Plate Electrode Experiment

To explore the mechanism of LM droplet driving, an additional experiment was performed with a large plate electrode. The large plate electrode was made of a thin copper sheet (1.0 cm × 0.5 cm) with a silver-plated copper wire welded to it, as shown in Figure 1c. For the convenience of the stable placement of the plate electrode, the copper sheet was stuck to a 4 mm thick PDMS block. In order to prevent direct contact between the electrode and the liquid metal, the surface of the copper sheet was spin-coated with a 50 micron thick PDMS membrane. 

**Figure 5:**
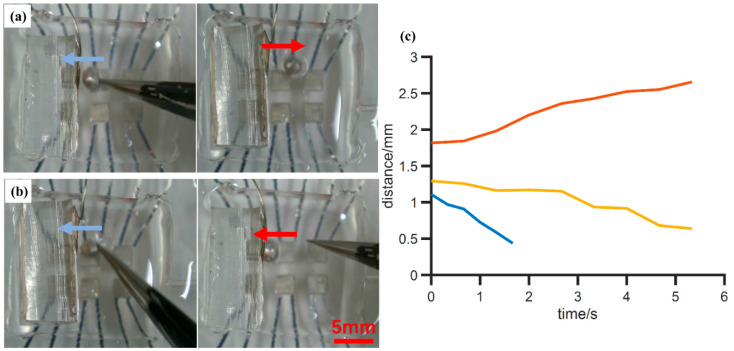
Experimental results of LM droplet moving towards a copper board electrode. Photo of droplet’s performance when it is further from (**a**) or nearer (**b**) the copper sheet electrode (+1000 V). (**c**) Test of droplet movement with different initial distance. Different colored lines represent different tests. *x*-axis: recording time; *y*-axis: distance between droplet center and board electrode. Zero time point is voltage application time.

This experiment aims to see how an LM droplet is driven under different electric field strengths. During the experiment, the PDMS-attached plate electrode was put vertically in the manipulation pool, as shown in Figure 5. A +1000 V D.C. voltage was applied on the plate electrode, and the electrodes in the DEP microchip remained unpowered. An LM droplet was put into the manipulation pool and its position was manually controlled with a pair of tweezers. Experimental results will be presented later.

## 3. Results

This section will be divided into two parts. It provides a concise and precise description of the experimental results, their interpretation, as well as the experimental conclusions that can be drawn. 

In the first part of the experiment, the performance of this LM droplet driving device was tested. Voltages were applied on six electrodes on the microchip, labeled as “a” to “f”, and a droplet with 2.0 mm diameter, which is the same size as the electrode side length, was controlled to move as “S” tracks by a designed voltage sequence, as shown in Figure 2. The “+” and “-” signs in the figure represent +1000 V and −1000 V, respectively. The results show that the LM droplet tends to be attracted to the “−” voltage electrode. In the beginning, the electrode “a” was in “−” state, while other electrodes were in “+” state, and the LM droplet was attracted and trapped upon the electrode “a”, as shown in Figure 2a. In the second step, when the electrode “a” turned back to the “+” state, and the electrode “d” to the right of the electrode “a”, turned to the “−” state, the droplet moved to the right and was trapped upon the electrode “d”, as shown in Figure 2b. By turning the electrodes “e”, “b”, “c”, “f” to the “−” state, in turn, the droplet was controlled to move upon these electrodes by turns, and its track formed an opposite “S”. The whole experimental process was recorded with a camera. The LM droplet driving velocity is then calculated by calculating the time period while the droplet traveled between the two electrodes from the Video S1 (in mp4 format, liquid metal droplet manipulation process), and with the known distance, the velocity can be acquired. At the voltage of ±1000 V, the velocity is 0.5 mm/s. 

To study how the droplet size affects its movement velocity, a 1.0 mm diameter droplet, which is much smaller than the electrode size, was further tested, and the results are shown in Figure 3. All experimental conditions were the same as mentioned above. The result indicates that the smaller droplet moves faster than larger ones under the same voltage, in this case, the 1.0mm diameter droplet is driven with 1.0 mm/s velocity. However, smaller droplets would only move across the nearest electrode edges. As shown in Figure 3a, where the electrode “a” was “−” at the beginning, the LM droplet was trapped at the edge of the electrode and unable to go to the electrode center like the 2.0 mm diameter droplet. In the second step, when the electrode “a” was turned back to “+” state and “b” to the “−” state, the small droplet was driven to move towards the electrode “b”, and finally stopped at the electrode edge, as shown in Figure 3b,c. When the electrodes “a” and “b” turned back to “−” and “+”, respectively, again, the small droplet was driven back to the electrode “a”, and again, stabled at the electrode edge, as shown in Figure 3d,e. This indicates that the edges of the electrode provide larger driving forces or electric field gradients than the electrode inner area could. In actual application of this droplet driving device, the size of droplet and electrode should be reasonably chosen according to the actual need. 

In addition to the driving velocity, the way of the droplet movement is worth studying. During the process of the movement of the LM droplets between the bottom electrodes, LM droplets were found to move in a rolling manner, rather than a translative manner, as shown in Figure 4. This phenomenon indicates that the upper and lower parts of a droplet are subjected to forces in different directions under the electric field. The calculated rotation speeds for the 2 mm and 1 mm diameter droplets are 4.78 rpm and 19.11 rpm, respectively. A detailed analysis will be discussed later. 

Except for the 1000 V driving voltage, the performance of this device to other voltages were tested. The lowest voltage can be also seen as an effective voltage in the system and was found to be +1000 V/−500 V (and reversed). When the voltages went up to +2500 V/−2500 V, because the interval between neighboring copper wires (those used to connect the electrode on the device to the outer high voltage source) is about 2 mm at the outlets of the electrodes, the air between these two wires was broken down and a short circuit happened, and the whole device was invalidated. So, the range of voltages that can be used for this experiment is +1000 V/−500 V (and reversed) to ±2500 V. 

To further study the driving mechanism of the LM droplet driving, a driving experiment based on the copper sheet plate electrode was performed. During the experiment, a 2.0 mm diameter LM droplet was put into the manipulation pool, and the PDMS-attached plate electrode was put vertically in the manipulating pool, as shown in Figure 5. The six “island” electrodes beneath the manipulation pool were not connected to any power supply and remained at a floating voltage. A +1000 V voltage was applied to the copper sheet electrode. As shown in Figure 5a, when the LM droplet stayed relatively far from the copper sheet and was slowly pushed against the copper sheet manually by a pair of tweezers, the droplet would spontaneously move away from the copper electrode. However, when the droplet was pushed harder and got closer to the electrode, the droplet would suddenly be attracted by the electrode and voluntarily moved towards and finally “stuck” to the copper electrode, as shown in Figure 5b. We quantified this process by repeating the experiment with different initial distances between the electrode and droplet, and the results are shown in Figure 5c, where the *y*-axis indicates the distance between electrode interface and the center of the droplet, and the *x*-axis indicates the recording time. The zero-time point is the time when voltage is applied on an electrode, and the distance at this point represents the initial position of the droplet. In the three repeats shown here, when the droplet was initially set nearer to the electrode, as shown in yellow and blue lines, it would be attracted and finally stuck to the electrode. The shorter the initial distance was, the faster the droplet moved. If the initial distance was larger, like the one shown by the red line, the droplet was pushed away from the electrode. After sticking to the electrode, the shape of the LM droplet was changed from a ball to an ellipsoid, as shown in Figure 5b. So, the final distance between the droplet center and the electrode could be smaller than the droplet radius. This phenomenon might be explained by the fact that the droplet induces different charge distributions when it is at different distances from the electrode. The details will be provided in Section 4. 

## 4. Discussion

In this study, a DEP device was developed and tested for driving liquid metal (LM) droplets. Directional movements of a LM droplet in this work were achieved by applying DEP forces. By controlling the electrode field direction around those droplets, the LM droplets were driven with a velocity of 0.5 mm/s for 2.0 mm-diameter droplets, and 1.0 mm/s for 1.0 mm-diameter droplets at 1000 V. Theoretical analysis of the driving phenomena will be discussed below. This LM droplet driving device is made of PDMS, where laser ablation was used in its microchannel fabrication process. Room temperature liquid metal, gallium-based alloy (Ga_66_In_20.5_Sn_13.5_) is used as electrode material in this device. In all, this device drives LM droplets by DEP forces, giving an alternative in liquid metal droplet manipulation, and has potential in microfluidics devices like pumps, valves, and soft robots. 

**Figure 6:**
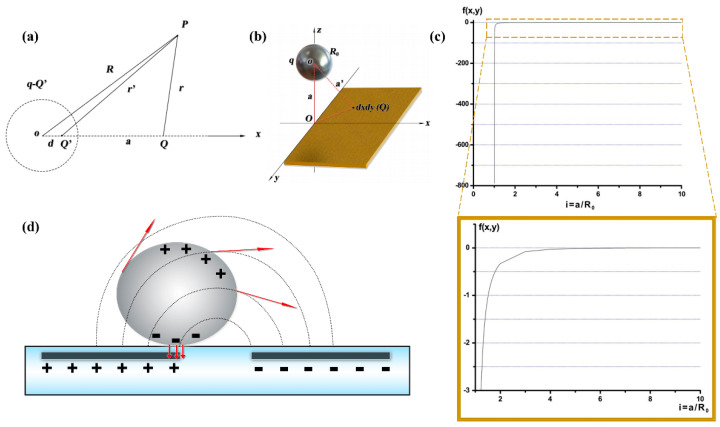
(**a**) Geometrical model of theoretical analysis; (**b**) geometric model of copper board electrode and LM droplet; (**c**) calculation result of forces on LM droplet from charged board with different droplet-board distance; (**d**) induced charge in a LM droplet inside electric field and schematic of droplet rolling principle.

First, to explore the theoretical basis of this LM droplet driving device, a simplified numeral model is established. According to the force analysis of the charge in the electrostatic field, the potential value of any point P in space should be found when there exists an ungrounded metal ball with radius $R_{0}$ and charge q and a point charge *Q*, as shown in Figure 6a. The ball is not grounded, so that charges are induced by the point charge *Q*. According to the mirror image method, the induced charge on the ball can be equivalently expressed as an image charge $Q^{\prime }$. As shown in Figure 6a, according to reference [30], the electric potential $\varphi_{0}$ at *P* can be expressed as:

(1) $$\varphi_{0}=\frac{1}{4\pi \varepsilon_{0}}\left\{\frac{Q}{{\left(R^{2}+a^{2}-2Racos\theta \right)}^{2}}-\frac{\frac{R_{0}}{a}Q}{{\left(R^{2}+\frac{R_{0}^{4}}{a^{2}}-2R\frac{R_{0}^{2}}{a}cos\theta \right)}^{\frac{1}{2}}}+\frac{q+\frac{R_{0}}{a}Q}{R}\right\}$$

where a is distance between the image charge and the point charge *Q*, R is distance between the point P and the center of the ball. The image charge $Q^{\prime }$ is expressed as:

(2) $$Q^{\prime }=-\frac{R_{0}Q}{a}$$

The force between the metal ball and the charge Q is calculated by:

(3) $$\vec{F}=Q\vec{E}$$

where

(4) $$\vec{E}=-\frac{\partial \varphi_{0}}{\partial n}\hat{\vec{n}}{|}_{R=a}=-\frac{\partial \varphi_{0}}{\partial R}{\vec{e}}_{x}{|}_{R=a}$$

Combining Equations (1)–(4), $\vec{F}$ can be expressed as:

(5) $$\vec{F}=\frac{1}{4\pi \varepsilon_{0}}\left[\frac{Q\left(q+\frac{R_{0}}{a}Q\right)}{a^{2}}-\frac{Q^{2}\frac{R_{0}}{a}}{{\left(a^{2}-\frac{R_{0}^{2}}{a}\right)}^{2}}\right]{\vec{e}}_{x}=\frac{1}{4\pi \varepsilon_{0}}\frac{Q}{a^{2}}\left[q-\frac{QR_{0}^{3}\left(2a^{2}-R_{0}^{2}\right)}{a{\left(a^{2}-R_{0}^{2}\right)}^{2}}\right]{\vec{e}}_{x}$$

Then, Equation (5) can be used to derive the electric force exerted on the metal ball by a plate electrode. The point charge Q can be regarded as the charge of a small element dxdy on the plate electrode, as shown in Figure 6b. According to Newton’s third law, the metal ball is also subjected to an opposing force $-\vec{F}$ from Q. Thus, to calculate the total force acting on the ball for a given area of the plate electrode, $\vec{F}$ can be integrated as follows:

(6) $${\vec{F}}_{all}=\iint \frac{1}{4\pi \varepsilon_{0}}\frac{Q}{{\left(\sqrt{x^{2}+y^{2}+a^{2}}\right)}^{2}}\left\{q-\frac{QR_{0}^{3}\left[2{\left(\sqrt{x^{2}+y^{2}+a^{2}}\right)}^{2}-R_{0}^{2}\right]}{\sqrt{x^{2}+y^{2}+a^{2}}\left[{\left(\sqrt{x^{2}+y^{2}+a^{2}}\right)}^{2}-R_{0}^{2}\right]}\right\}dxdy$$

Equation (6) can be used to calculate the force applied on the LM droplet from a charged plate electrode. It is assumed that it is a ball-shaped droplet with radius 1.0 mm. The size of the charged plate is similar to the copper sheet used in the experiment (0.5 cm × 1.0 cm). Assuming $b=q/Q,\;i=a/R_{0}$, Equation (6) can be written as:

(7) $${\vec{F}}_{all}=\frac{Q^{2}}{4\pi \varepsilon_{0}}\int_{y_{1}}^{y_{2}}\int_{x_{1}}^{x_{2}}f\left(x,y\right)dxdy$$

where

(8) $$f\left(x,y\right)=\frac{1}{{\left(\sqrt{x^{2}+y^{2}+a^{2}}\right)}^{2}}\left\{b-\frac{R_{0}^{3}\left[2{\left(\sqrt{x^{2}+y^{2}+a^{2}}\right)}^{2}-R_{0}^{2}\right]}{\sqrt{x^{2}+y^{2}+a^{2}}\left[{\left(\sqrt{x^{2}+y^{2}+a^{2}}\right)}^{2}-R_{0}^{2}\right]}\right\}$$

The result of ${\vec{F}}_{all}$ is plotted in Figure 6c. In the experiment, the LM droplets were uncharged, so b=0. When i≤2, ${\vec{F}}_{all}$ is negative, meaning that the force on the droplet is attractive when the droplet is close to the plate electrode. As i becomes smaller, ${\vec{F}}_{all}$ decreases sharply, indicating the attractive force on the droplet becomes larger as well. As i becomes larger, ${\vec{F}}_{all}$ slowly increases and finally becomes positive, and forces on the droplet become repulsive accordingly. 

During the experiment, when the LM droplet was pushed to the charged copper sheet, repulsive forces were first observed when the distance between the droplet and the copper sheet was large. This is in good agreement with the calculation results with larger i. When the droplet was pushed closer, the repulsive force turned into an attractive force, and the droplet spontaneously moved towards the charged electrode, until they stuck together. This is similar to the calculation results with smaller i. So, the theoretical analysis and the experimental results are consistent. 

As described in the former part, when moving between the square electrode paddles, the droplet moved in a rolling manner, instead of a translative manner. This could be explained by the fact that the droplet is driven by induced charges in the electric field from the electrodes below. As shown in Figure 6d, when the LM droplet was put into an electric field, charges inside the droplet would move according to the electric field. Take the electric field in Figure 6d as an example, the negative charges inside the droplet would be attracted to the bottom by the positive electrode beneath it; positive charges inside the droplet will then be repelled to the upper part of the droplet. As shown in Figure 6d, when a LM droplet was placed between two opposite electrodes, the upper part of the droplet was attracted to the electrode ahead and started to move forward, while the lower part, with negative charge induced, was attracted to the electrode beneath it, and tended to stay still. Thus, the droplet would roll forward instead of sliding forward. 

In most reported LM droplet manipulation studies, using electricity to control is the most convenient method. In these devices, LM droplets, not only gallium-based alloys, but mercury as well, are driven by a pair of electrodes to move or transform to perform as switches, pumps, or mixers [19,20]. In most cases, LM droplets have to be wholly wrapped with electrolyte solution, such as NaOH solution, so that the droplet could be easily electrically driven [29,30]. In the DEP device proposed in this work, on the other hand, no electrolyte is necessary. A thin layer of silicone oil, as mentioned above, is applied in the manipulation pool, simply to prevent an oxide film on gallium-based alloy droplets from sticking to PDMS. Due to the high viscosity (350 ± 10 mPa·s at 25 °C) and wettability of silicone oil to PDMS, there would be a very thin trace of oil left when it flows through the PDMS surface of the manipulation pool. This layer is thick enough to prevent LM droplets from sticking to the PDMS surface, but thin enough to maintain low flow resistance during LM droplet movement. Besides, in this DEP device, there is no need to surround the droplet with fluid, like silicone oil or electrolyte solution; LM droplets could be driven in air, and only a thin protection layer on the PDMS surface is needed. The device proposed here gets rid of this limitation and shows strong potential in wider applications of LM droplet movement. 

As for metal droplet driving studies based on the electrostatic principle, contact electrodes are used in most cases, i.e., electrodes generating an electric field are in direct contact with the LM droplet itself, or with electrolyte around the droplet. Except for sample contamination from contact electrodes, bubble generation and heating problems during use are of more concern. The electrode materials involved in this kind of device are mainly inert metals, like gold or platinum, to prevent their reaction with electrolyte and electrode corrosions. However, high voltage and current applied on electrodes while working, would cause hydrolysis, and bubbles performed from hydrolysis might block microchannels and invalidate the whole device; the occurrence of squeezing bubbles needs additional channel design or surface modification of the microchannel walls. To avoid bubble formation, voltages used in electrostatic droplet manipulation are limited, so that the driving efficiency is restricted. In the device mentioned in this work, there is a thin PDMS membrane between the droplet and the driving electrode, thereby avoiding any contact of the electrodes with the working droplet. Thus, all problems that might happen in contact-electrode devices are perfectly prevented. 

Except for removing the restriction of electrolyte solution during manipulation and application of non-contact electrodes, this device is advantageous over currently existing LM droplet manipulation platforms by its flexible electrode design. In most reported platforms for LM droplet control, only one pair of electrodes is used, so that the moving route of an LM droplet is restricted, mostly controlled by the shape of microchannels; usually one structure could provide only one motion path for droplets. In the device reported here, on the other hand, a multi-electrode structure is used. The position and moving routes of droplets are controlled by the electric field provided from electrodes, so that this device could realize a series of moving paths for droplets within one design. Electrodes are set beneath the manipulation pool, making it possible to bring more complicated electrode patterns to this device, and to realize more complicated and flexible movement patterns of LM droplets than most of the reported works.

The simplest and most direct application of this device is a surface microswitch for electrical, thermal, or even optical control. With proper electrode design and applied voltage sequence, LM droplets could be controlled to move to the needed position to “open” or “close” a circuit or heater. With more design, this device could also be used to build valves, pumps, or even soft robots. However, due to the low electrical conductivity of PDMS, the driving voltage of this device is much higher than previously reported electrical devices. Reducing the thickness of the PDMS membrane between electrodes and droplets could reduce the voltage needed in droplet driving. Except for the driving voltage, more attempts are expected with this design and driving principle in the future. 

## 5. Conclusions

Here, a microfluidic device for liquid metal (LM) droplet manipulation is presented, tested, analyzed, and discussed. By applying different voltages on six square electrodes in this device, a non-uniform electric field is formed inside the manipulation pool; LM droplets inside the pool are then induced and partially charged, and driven according to the electric field. With ±1000 V voltages applied on electrodes, LM droplets are driven 0.5 mm/s for 2.0 mm-diameter ones, and 1.0 mm/s for 1.0 mm-diameter ones. The whole chip is formed by PDMS, and microchannel structures are fabricated by laser ablation, a simpler and alternative approach compared to standard soft lithography. In this device, there is a thin PDMS film between the electrodes and the manipulation pool, so that the electrodes are not in direct contact with the working droplets, preventing Joule heat or bubble formation during working. Gallium-based alloy with melting point 10.6 °C is used as electrode material in this device, to simplify the fabrication process. This DEP metal droplet driving device is expected to be a flexible approach for metal droplet driving.

## Supplementary Materials

The following are available online at https://www.mdpi.com/2072-666X/12/3/340/s1, Video S1: liquid metal droplet manipulation process.

## References

[1] Basova E.Y., Foret F. (2015). Droplet microfluidics in (bio)chemical analysis. Analyst.

[2] Mashaghi S., Abbaspourrad A., Weitz D.A., van Oijen A.M. (2016). Droplet microfluidics: A tool for biology, chemistry and nanotechnology. Trends Anal. Chem..

[3] Teh S.Y., Lin R., Hung L.H., Lee A.P. (2008). Droplet microfluidics. Lab Chip.

[4] Shang L., Cheng Y., Zhao Y. (2017). Emerging droplet microfluidics. Chem. Rev..

[5] Roux J., Fouillet Y., Achard J. (2007). 3D droplet displacement in microfluidic systems by electrostatic actuation. Sens. Actuators A Phys..

[6] Ahn B., Lee K., Louge R., Oh K.W. (2009). Concurrent droplet charging and sorting by electrostatic actuation. Biomicrofluidics.

[7] Nelson W.C., Kim C.J.C. (2012). Droplet actuation by electrowetting-on-dielectric (EWOD): A review. J. Adhes. Sci. Technol..

[8] Wang K.L., Jones T.B., Raisanen A. (2007). Dynamic control of DEP actuation and droplet dispensing. J. Micromech. Microeng..

[9] Ahmed R., Jones T.B. (2007). Optimized liquid DEP droplet dispensing. J. Micromech. Microeng..

[10] Joensson H.N., Svahn A.H. (2012). Droplet microfluidics—A tool for single-cell analysis. Angew. Chem. Int. Ed..

[11] Tenje M., Fornell A., Ohlin M., Nilsson J. (2018). Particle manipulation methods in droplet microfluidics. Anal. Chem..

[12] Shui L., Eijkel J.C.T., van den Berg A. (2007). Multiphase flow in microfluidic systems—Control and applications of droplets and interfaces. Adv. Colloid Interface Sci..

[13] Zhu L., Wang B., Handschuh-Wang S., Zhou X. (2020). Liquid metal-based soft microfluidics. Small.

[14] Liu R., Yang X., Jin C., Fu J., Chen W., Liu J. (2013). Development of three-dimension microelectrode array for bioelectric measurement using the liquidmetal-micromolding technique. Appl. Phys. Lett..

[15] Wang Q., Yu Y., Yang J., Liu J. (2015). Fast Fabrication of flexible functional circuits based on liquid metal dual-trans printing. Adv. Mater..

[16] Sun X., Yuan B., Rao W., Liu J. (2017). Amorphous liquid metal electrodes enabled conformable electrochemical therapy of tumors. Biomaterials.

[17] Yi L., Jin C., Wang L., Liu J. (2014). Liquid-solid phase transition alloy as reversible and rapid molding bone cement. Biomaterials.

[18] Sen P., Kim C.J. (2009). A fast liquid-metal droplet microswitch using EWOD-driven contact-line sliding. J. Microelectromech. Syst..

[19] Yang T., Kwon B., Weisensee P.B., Kang J.G., Li X., Braun P., Miljkovic N., King W.P. (2018). Millimeter-scale liquid metal droplet thermal switch. Appl. Phys. Lett..

[20] Kim J., Shen W., Latorre L., Kim C. (2002). A micromechanical switch with electrostatically driven liquid-metal droplet. Sens. Actuators A Phys..

[21] Tang S., Khoshmanesh K., Sivan V., Petersen P., Mullane A.P.O., Abbott D. (2014). Liquid metal enabled pump. Proc. Natl. Acad. Sci. USA.

[22] Hu Q., Ren Y., Liu W., Chen X., Tao Y., Jiang H. (2017). Fluid flow and mixing induced by AC continuous electrowetting of liquid metal droplet. Micromachines.

[23] Wu J., Tang S., Fang T., Li W., Li X., Zhang S. (2018). A wheeled robot driven by a liquid-metal droplet. Adv. Mater..

[24] Pethig R. (2010). Dielectrophoresis: Status of the theory, technology, and applications. Biomicrofluidics.

[25] Tian L., Gao M., Gui L. (2017). A microfluidic chip for liquid metal droplet generation and sorting. Micromachines.

[26] Çetin B., Li D. (2011). Dielectrophoresis in microfluidics technology. Electrophoresis.

[27] Wang R., Zhang L., Gao M., Wang Q., Deng Z., Gui L. (2019). A liquid-metal-based dielectrophoretic microdroplet generator. Micromachines.

[28] Zhang L., Zhang P., Wang R., Zhang R., Li Z., Liu W., Wang Q., Gao M., Gui L. (2020). A performance-enhanced liquid metal-based microheater with parallel ventilating side-channels. Micromachines.

[29] Ye Z., Zhang R., Gao M., Deng Z., Gui L. (2019). Development of a high flow rate 3-D electroosmotic flow pump. Micromachines.

[30] Guo S. (1980). Electrodynamics.

